# 30 year experience of index case identification and outcomes of cascade testing in high-risk breast and colorectal cancer predisposition genes

**DOI:** 10.1038/s41431-021-01011-8

**Published:** 2021-12-06

**Authors:** Emma R. Woodward, Kate Green, George J. Burghel, Michael Bulman, Tara Clancy, Fiona Lalloo, Helene Schlecht, Andrew J. Wallace, D. Gareth Evans

**Affiliations:** 1 Manchester Centre for Genomic Medicine, Manchester University Hospitals NHS Foundation Trust, Manchester, M13 9WL UK; 2 Division of Evolution and Genomic Sciences, School of Biological Sciences, Faculty of Biology, Medicine and Health, University of Manchester, Manchester Academic Health Science Centre, Manchester, M13 9PL UK

**Keywords:** Cancer genetics, Genetics research

## Abstract

It is 30 years since the first diagnostic cancer predisposition gene (CPG) test in the Manchester Centre for Genomic Medicine (MCGM), providing opportunities for cancer prevention, early detection and targeted treatments in index cases and at-risk family members. Here, we present time trends (1990–2020) of identification of index cases with a germline CPG variant and numbers of subsequent cascade tests, for 15 high-risk breast and gastro-intestinal tract cancer-associated CPGs: *BRCA1*, *BRCA2*, *PALB2*, *PTEN*, *TP53*, *APC*, *BMPR1a*, *CDH1*, *MLH1*, *MSH2*, *MSH6*, *PMS2*, *SMAD4*, *STK11* and *MUTYH*. We recorded 2082 positive index case diagnostic screening tests, generating 3216 positive and 3140 negative family cascade (non-index) tests. This is equivalent to an average of 3.05 subsequent cascade tests per positive diagnostic index test, with 1.54 positive and 1.51 negative non-index tests per family. The CPGs with the highest numbers of non-index positive cases identified on cascade testing were *BRCA1/2* (*n* = 1999) and the mismatch repair CPGs associated with Lynch Syndrome (*n* = 731). These data are important for service provision and health economic assessment of CPG diagnostic testing, in terms of cancer prevention and early detection strategies, and identifying those likely to benefit from targeted treatment strategies.

## Introduction

The identification of the *TP53* gene as a high-risk Cancer Predisposition Gene (CPG) in 1990, followed in rapid succession by the *APC*, *BRCA1*, *BRCA2* and Lynch (formerly HNPCC) genes in 1991–1995, enabled the introduction of CPG testing into the cancer genetics clinic.

The detection of a high-risk germline CPG variant in an index case provides not only cancer prevention and treatment opportunities in that individual but, through family cascade testing, subsequent cancer prevention and early detection opportunities in at-risk family members testing positive. Family members testing negative can, for the most part, be released from high-risk screening programmes, relieving anxiety and, for some CPGs such as *APC*, sparing young teenagers from invasive investigations.

The Manchester Centre for Genomic Medicine (MCGM) serves a population of five million in the North West of England. Outcomes of clinical CPG testing in the clinic, both diagnostic and predictive, have been recorded by the MCGM through the use of family registers since diagnostic CPG testing first started in 1990 [1, 2]. This record provides a clinical tool to ensure appropriate screening is in place, family members are offered predictive genetic testing as appropriate, and provides a resource for identifying patients when new management approaches or research opportunities become available.

As it is now 30 years since the first diagnostic CPG test, and a personal/family history of breast (68%), ovarian (12%) and colorectal cancers (18%) are the most common referral reason to clinical genetics [3], we sought to evaluate, for these CPGs, time trends of variant detection in index cases, and numbers of cascade tests undertaken. Numbers and outcomes of subsequent cascade tests across these CPGs have not, to our knowledge been investigated before, a figure that is useful for downstream resource planning.

## Methods

Genetic registers were interrogated for numbers of diagnostic and subsequent family cascade tests (positive and negative), and years of testing, in 15 high-risk CPGs associated with breast and gastro-intestinal (GI) tract cancers. The CPGs interrogated were: *BRCA1*, *BRCA2*, *PALB2*, *PTEN*, *TP53*, *APC*, *BMPR1a*, *CDH1*, *MLH1*, *MSH2*, *MSH6*, *PMS2*, *SMAD4*, *STK11* and *MUTYH*. Data pertaining to families with Familial Adenomatous Polyposis Coli (FAP) investigated solely through linkage studies is not presented, nor is that pertaining to germline *MLH1* hypermethylation, nor individuals where mosaicism for a CPG variant was identified. Only class 4 and class 5 CPG variants are presented and are referred to, collectively, as “variants” [4, 5]. Bi-allelic *MUTYH* cases are included. Testing of moderate-risk genes such as *CHEK2* and *ATM* has never been comprehensive and was excluded. Data were censored on 6th October 2020. Of note, there was much-reduced capacity for both laboratory testing and clinic appointments for much of 2020 in view of the SARS-CoV2 pandemic.

## Results

### Timeline of testing and strategies

The introduction of diagnostic testing of CPGs in the MCGM began in the early 1990s mirroring the early days of CPG identification when testing was initially undertaken on a research basis prior to formal incorporation into the clinical diagnostic setting in 1998. Thus, testing began with *TP53* in 1990 following its identification as being causative of Li Fraumeni Syndrome in 1990 [6], followed by *APC* in 1993, then *BRCA1* in 1994 and *BRCA2*, *MLH1* and *MSH2* in 1996. The latest CPG to be included in clinical diagnostic testing in the MCGM was *PALB2* in 2016; although having been identified as causative of hereditary breast cancer in 2007 [7], it was not introduced until later studies confirmed its role as a high-risk breast cancer CPG [8, 9].

Initial PGV detection strategies were by SSCP, dHPLC and Southern Blot analyses, prior to the introduction of Sanger sequencing in 2003, MLPA for copy number detection in 2005/2006 and next-generation sequencing (NGS) in 2014 (2013 for *BRCA1/2*).

### Subsequent non-index cascade tests

Since 1990 there have been a total of 2082 positive index case diagnostic tests. Over the 30-year time span of germline genetic testing, these positive diagnostic tests have generated a total of 3216 positive family cascade (non-index) tests and 3140 negative non-index tests, equivalent to an average across all 15 CPGs investigated of 3.05 subsequent cascade tests per positive diagnostic test (Table 1). Subsequent cascade tests per family ranged from <1.0 for *MUTYH* (*n* = 0.15) and *PTEN* (*n* = 0.62), to >3.0 for *BRCA1* (*n* = 3.06), *BRCA2* (*n* = 3.40), *MLH1* (*n* = 3.35) and *MSH2* (*n* = 3.02).

**Table 1:** Index and cascade testing for high-risk breast and GI tract cancer predisposition genes in MCGM, 1990–2020.

Gene	Year first positive index	Total positive index cases (*N*)	Total positive non-index cases (*N*)	Rate of cases per year (index & non-index)	Total negative non-index cases (*N*)	Non-index tests per index case detected	Positive non-index tests per index case detected	Negative non-index tests per per index case detected	Ratio non-index positive tests/non-index tests per index case detected
*BRCA1*	1994	644	975	59.96	994	3.06	1.51	1.54	0.50
*BRCA2*	1996	592	1024	64.64	986	3.40	1.73	1.67	0.49
*PALB2*	2016	23	28	10.20	11	1.70	1.22	0.48	0.72
*PTEN*	2001	21	13	1.70	0	0.62	0.62	0.00	1.00
*TP53*	1990	62	79	4.55	48	2.05	1.27	0.77	0.62
*APC*	1993	180	314	17.64	252	3.14	1.74	1.40	0.55
*BMPR1a*	2008	7	11	1.38	3	2.00	1.57	0.43	0.79
*CDH1*	2004	7	13	1.18	10	3.29	1.86	1.43	0.57
*MLH1*	1996	162	234	15.84	309	3.35	1.44	1.91	0.43
*MSH2*	1996	208	301	20.36	328	3.02	1.45	1.58	0.48
*MSH6*	2002	92	126	11.47	131	2.79	1.37	1.42	0.49
*PMS2*	2008	45	70	8.85	54	2.76	1.56	1.20	0.44
*MUTYH1*^a^	2002	20	3	1.21	0	0.15	0.15	0.00	1.00
*SMAD4*	1999	9	10	0.86	7	1.89	1.11	0.78	0.59
*STK11*	2001	10	15	1.25	7	2.20	1.50	0.70	0.68
Total	N/A	2082	3216	N/A	3140	3.05	1.54	1.51	0.51

^a^bi-allelic cases only included.

Across all 15 CPGs there was an almost equitable division of the number of positive (*n* = 1.54) and negative (*n* = 1.51) subsequent cascade tests per index case identified, although some of these were confirmatory tests of relatives with appropriate cancer or phenotype (e.g., breast or ovarian cancer for *BRCA1/2* or typical Cowden features for *PTEN*). CPGs where the ratio of subsequent positive tests/total subsequent tests was >0.6 included *PALB2* (*n* = 0.72), *TP53* (*n* = 0.62), *BMPR1a* (*n* = 0.79), *STK11* (*n* = 0.68), *MUTYH* (*n* = 1.00) and *PTEN* (*n* = 1.00). None of the CPGs investigated had a ratio of subsequent positive tests/total subsequent tests <0.4 (Table 1).

As *BRCA1* and *BRCA2* were found to account for over half of the index and subsequent cascade tests in our centre (*BRCA1/2* index 1236/2082 = 59.37%; *BRCA1/2* non-index 3979/6356 = 62.60%, Table 1), we then sought to investigate the numbers of non-index tests by family. This showed the minimum number of cascade tests in a family for both *BRCA1* and *BRCA2* was zero; for *BRCA1* the maximum number of non-index tests in a family was 25, and 74 for *BRCA2* (Supplementary Table 1).

### Time trends for the common CPGs

**Fig. 1:**
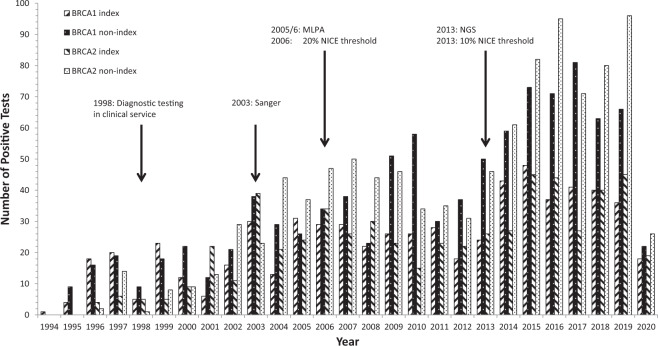
*BRCA1* and *BRCA2* index and non-index case positive tests by year. Bar chart showing, for each year of testing, numbers of the index and non-index cases identified for each of *BRCA1* and *BRCA2*. MLPA multiplex ligation-dependent probe amplification, NICE National Institute for Health and Care Excellence, NGS next-generation sequencing.

Diagnostic genetic testing for *BRCA1* began in 1994 with the finding of one index case germline variant followed by four further index cases in 1995 and nine non-index cases, reaching a maximum of 48 index cases identified in 2015 and 81 non-index cases in 2017 (Fig. 1). For *BRCA2*, the first index case germline variants were identified in 1996 (*n* = 4) along with two non-index cases, reaching a maximum of 45 index cases identified in both 2015 and 2019, and a maximum of 95 non-index cases in 2016 and 96 in 2019 (Fig. 1).

**Fig. 2:**
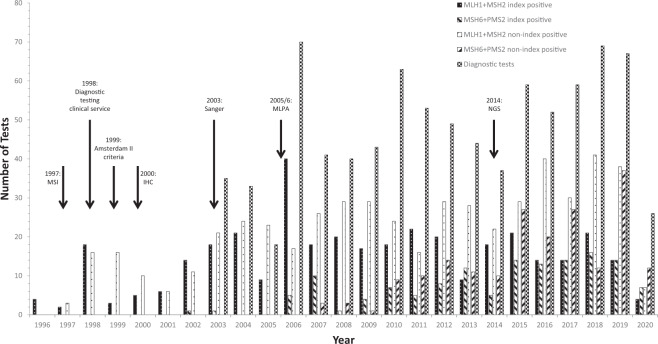
MLH1/MSH2 and MSH6/PMS2 index and non-index case positive tests by year and total numbers of diagnostic tests. Bar chart showing, for each year of testing, numbers of the index and non-index cases identified for *MLH1*, *MSH2* and *MSH6*, *PMS2*. Arrows denote changes to diagnostic testing strategies. Data for the total number of annual diagnostic tests undertaken is from 2003. MSI microsatellite instability (studies); IHC immunohistochemistry.

**Fig. 3:**
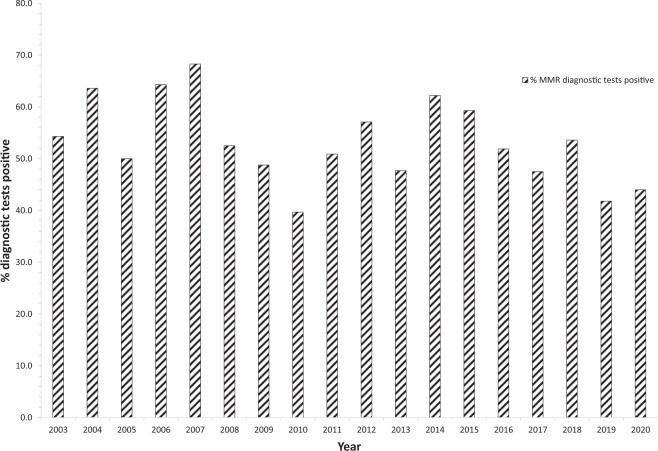
Percentage of diagnostic mismatch repair (MMR) genetic tests where a variant was detected. Bar chart showing, for each year of testing from 2003 when Sanger sequencing was introduced, the percentage of diagnostic MMR tests where a variant was detected.

For the mismatch repair (MMR) CPGs, testing began in 1996 with *MLH1* and *MSH2* genes where four (*MLH1*, *n* = 1; *MSH2*, *n* = 3) index cases were identified, followed in 1997 with the identification of three non-index cases (*MLH1*, *n* = 1; *MSH2*, *n* = 2) (Fig. 2). The first *MSH6* index case was identified in 2002 (*n* = 1) and for *PMS2* in 2008 (*n* = 1). We were able to attain numbers of diagnostic MMR tests undertaken from 2003 onwards when Sanger sequencing was first introduced and, from this calculate the percentage of diagnostic tests in which a CPG variant was detected (mean = 53.2%, 95% CIs = 49.2–57.2%, median = 52.2%, minimum = 39.7%, maximum = 68.3%) (Figs. 2 and 3).

**Fig. 4:**
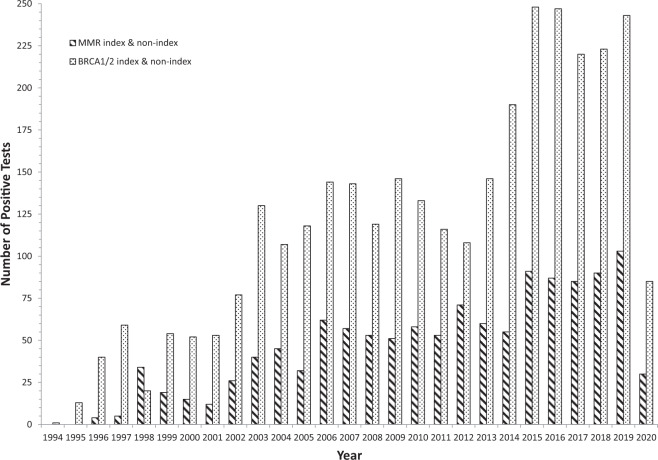
MMR and *BRCA1/2* index and non-index case positive tests by year. Bar chart showing, for each year of testing, the comparison of total numbers of the index and non-index cases identified for the MMR CPGs and *BRCA1*/*BRCA2*.

Considering the most commonly altered CPGs, a total of 1236 *BRCA1/2* index cases and 1999 non-index cases, and 507 MMR index cases and 731 non-index cases were identified since testing began in 1994 and 1996 respectively (Table 1, Fig. 4). Time trends have shown numbers of cases to increase over the testing period with over 2.6-fold greater *BRCA1/2* cases (index and non-index) identified as compared with MMR (*BRCA1/2* total cases, *n* = 3235; MMR total cases, *n* = 1238) (Table 1, Fig. 4).

### Cases (index and non-index) identified for each year of testing

As anticipated from the time-trend data, we found *BRCA2* and *BRCA1* to be the CPGs with the most variants (index and non-index cases) identified for each year of testing (*BRCA2*, *n* = 64.6; *BRCA1*, *n* = 60.0, per annum) (Table 1); whereas other CPGs had markedly less cases identified for each year of testing. CPGs with >10 cases identified per year of testing were *PALB2* (*n* = 10.2), *MSH6* (*n* = 11.5), *MLH1* (*n* = 15.8), *APC* (*n* = 17.7) and *MSH*2 (*n* = 20.4) (Table 1).

### GI tract CPGs-negative tests

The management of high-risk GI cancer screening through endoscopic surveillance [often coupled with risk-reducing surgery in FAP and hereditary diffuse gastric cancer (HDGC)], starting from as young as age 10–11 y in FAP and 18 y in HDGC, in individuals with an *APC* or *CDH1* variant has been universally accepted since clinical recognition of these conditions. This, coupled with the lack of known modifying risk factors sufficient to alter the screening protocol, means there is value in identifying non-carriers to avoid the necessity for, and potential anxiety around, this high-risk protocol. Therefore, we also considered the negative non-index cases in our high-risk GI tract patient cohort. Since the advent of *APC* testing in 1993, we have identified 252 non-index case negative individuals from 180 families (*n* = 1.4 negative tests for each index case identified) and, for the MMR genes, 822 negative non-index cases for the 507 families (*n* = 1.6 negative tests for each index case identified). A similar ratio was seen with *CDH1*, although lower for *BMPR1a* (three non-index negative tests from seven families, ratio = 0.4) (Table 1).

## Discussion

The provision of CPG testing has changed markedly and revolutionised the practice of clinical cancer genetics over the past 30 years. Where a CPG variant is identified in an index case this facilitates (i) management strategies in the index case, for example, decisions around the extent of surgical management or targeted therapeutic strategies (ii) cancer prevention and early detection strategies in at-risk family members and the index case where there is a second primary tumour risk [10].

The 1990s represented the zenith of high-risk CPG identification with 14/15 CPGs investigated in this study being identified during this time. For the subsequent years into the early 2000s prior to the introduction of Sanger sequencing and MLPA to detect deletions, the laborious and expensive nature of molecular investigations meant that variant detection was limited in sensitivity and there were strict eligibility criteria for testing.

Furthermore, in these early days, the main motivation for testing was to inform risk in family members as targeted therapeutic strategies such as PARP inhibitors where there is BRCA1/2 deficiency and PD-1 inhibitors for MMR-deficient tumours, was not available at this time, being dependent on the subsequent elucidation of the underlying molecular mechanisms of tumorigenesis [11, 12].

The situation in MCGM was similar both nationally and internationally. Therefore, consensus strategies were put in place to identify those individuals and families in whom there was the greatest likelihood of detecting a causative germline variant and who would most likely benefit from genetic investigations.

The original Amsterdam Criteria I [13] were devised initially to provide a consistent diagnosis of Lynch Syndrome (formerly known as HNPCC). Once germline genetic testing was available in the mid-1990s, the criteria were used to guide testing toward families most likely to harbour a germline variant in one of four MMR genes. Whilst being fairly specific, these criteria lacked sensitivity. They were amended in 1999 to allow the inclusion of both tumour testing strategies through MSI/IHC as a pre-screen, and extra-colonic cancers into the diagnostic criteria; this enabled more families to benefit from diagnostic CPG testing [14], with increasing numbers of index case CPG variants identified from this time onwards.

*PMS2* is worthy of particular mention. Direct sequencing is particularly challenging in view of its very close homology to *PMS2CL* [15, 16] with investigation often only offered where there is IHC loss of PMS2 protein and no germline alteration of *MLH1* detected. This would partly account for the overall fewer (*n* = 45) *PMS2* index cases detected as compared with the other MMR genes; however, a greater contribution to these lower numbers is likely to arise from the relatively low penetrance of *PMS2* (no increased cancer risk at <50 years of age in prospective studies of Lynch Syndrome) [17], despite *PMS2* variants being more common than for the other individual MMR genes [18] and further evidenced by the greater contribution to constitutional MMR deficiency [19].

The outcomes of initial *BRCA1/2* testing in Manchester lead to the development of the Manchester Score [20, 21] to identify individuals and families most likely to harbour a germline *BRCA1/2* variant. Points are given to cases of breast or ovarian cancer on the same side of a family, with additional points added or subtracted based on tumour pathology; a Manchester score of 15 equates to a 10% likelihood of detecting an underlying *BRCA1/2* variant and a score of 20 to a 20% likelihood. The threshold for clinical diagnostic testing was initially set at 20% [https://www.nice.org.uk/guidance/cg41] based on validated scoring techniques such as the Manchester score. This was decreased in 2013 to 10% following a NICE health economics assessment showing testing to be cost-effective in terms of impact on index cases and non-index family members (https://www.nice.org.uk/guidance/cg164/evidence/full-guideline-pdf-190130941), with a concomitant uplift in index cases detected. A further uplift in detection resulted from mainstreaming testing of all non-mucinous epithelial ovarian cancer from 2017 [22].

Our data showed overall more *BRCA1* than *BRCA2* index cases. This likely reflects the increased penetrance of BRCA1 as compared with *BRCA2* despite *BRCA2* alterations being more common in the population as exemplified by gnomAD data [gnomAD v2.1.1 accessed 15th February 2021 *BRCA1* loss of function (LoF), *n* = 141; *BRCA2* LoF, *n* = 248], control frequencies from the BRIDGES study [23] and a recent population study in the USA [18]. Germline *BRCA1* variants are often associated with young-onset life-limiting grade 3 triple-negative breast cancers [24], whereas for women ≥60 years with no previous cancer history who develop an epithelial ovarian cancer, *BRCA2* variants are more frequently detected [25].

Collectively, variants of the *BRCA1*, *BRCA2* and MMR CPGs are most common of all CPG variants, with an estimated population prevalence of 1 in 150 to 1 in 250 for *BRCA1/2* and 1 in 300 for Lynch [18, 23, 26]. We detected 2.4-fold more *BRCA1/2* index cases as compared with MMR index cases. This likely reflects breast cancer being much more common with over 55,000 new cases per annum (CRUK stats, https://www.cancerresearchuk.org/health-professional/cancer-statistics/statistics-by-cancer-type/breast-cancer accessed 6th November 2020), as compared with colorectal or endometrial cancer, and the consequent higher proportion of breast cancer referrals received to the MCGM, together with the higher penetrance of *BRCA1/2* variants as compared with those of the MMR CPGs.

As our data were primarily drawn from the MCGM registers where a CPG variant has been identified and we did not have access to all the diagnostic testing data, it is difficult to be certain of the index case detection rate and this was not the reason for this study. However, we do note a steady uplift in index cases with a CPG variant identified. As described above this likely reflects changes in both clinical criteria to put forward individuals and families for testing, and also advances in molecular strategies employed to enable CPG variant identification. Our data suggest that, in terms of molecular strategies, the introduction of Sanger sequencing in 2003, followed by MLPA in 2005/2006 to detect single exon to whole-gene deletions and duplications, did result in a significant uplift in index cases identified. Of note, previously we have shown whole-gene deletions to account for 14% of all germline CPG disease-associated variants [27]; prior to the introduction of MLPA their detection was laborious and of low sensitivity. Whilst the introduction of NGS in 2013/2014 has enabled much higher sample throughput with concomitant cost reduction, its introduction has not markedly increased the numbers of index cases identified, as shown by our MMR data where a total number of diagnostic tests undertaken from 2003 was known, with the 95% confidence intervals of variant detection residing within 49–57% throughout this period. Although NGS provides increased sensitivity for the detection of mosaicism as compared with conventional Sanger sequencing, its real utility as testing means primarily arises from cost reduction, speed and ability to simultaneously investigate multiple genes [28].

Across the 15 CPGs and 30 years of testing data we have shown that for each index case diagnosis attained, this leads to an average of three cascade tests within a family, with ~1.5 family members testing positive for the index case CPG variant. We were not able to find a previous assessment of this rate per index case detected; a figure that has importance for the health economic evaluation of index case diagnostic CPG testing, and has significant implications for cancer prevention strategies in at-risk family members.

Although our study was designed to investigate the overall numbers of CPG-positive index tests and subsequent cascade tests, we did explore the *BRCA1*/*2* uptake of cascade testing further as BRCA1/2 counts for >50% of the index and non-index tests in our centre. The only comparable study, albeit much smaller, was in the Dutch population [29], showing 50% uptake of cascade testing in family members for BRCA1/2. Overall, our at-risk population is much larger, and includes geographically disparate families so that not all cascade testing will be undertaken by our centre. Therefore, it is not possible to identify the exact numbers of at-risk family members in our population. However, within the genetics clinic, there is always the need for ongoing engagement with our families with a CPG variant, and this is facilitated in our centre by family registers and recall systems. The uptake of cascade testing at a family level in an individual centre will depend on many factors including the following: individual family size and structure; the clinical consequences of the CPG variant; whether there is cancer prevention and early detection intervention for family members testing positive; and geographical location of at-risk family members.

Where there were more positive than negative index cases identified, for the syndromic CPGs such as *STK11*, *BMPR1a* and *PTEN*, this may be a reflection of subsequent diagnoses in family members being made on clinical features and testing offered to confirm, with the absence of the phenotype not requiring genetic confirmation, particularly for *PTEN* and *STK11*. Considering *PALB2*, as diagnostic testing has only recently been introduced, the higher numbers of positive non-index cases may have occurred as genetic testing for other breast cancer predisposition genes may have already been undertaken in the family. Alternatively, these apparent higher numbers of positive non-index tests may be artefactual based on overall much smaller sample sizes arising from their relative rarity and fewer years of diagnostic testing. *MUTYH* is unusual in that bi-allelic variants cause *MUTYH* associated polyposis and only those cases are counted here which, for non-consanguineous families, will in practice be siblings only; again, the numbers are probably too small to draw direct conclusions from.

The value of a negative predictive test, especially for the high-risk GI cancer CPGs where there are not known to be other factors affecting management of cancer risk, is immense in terms of avoiding the need for invasive endoscopic investigations and potentially life-changing risk-reducing surgery from early teenage years/adulthood. Thus, it is notable that we have been able to reassure over 250 individuals from FAP families and over 20 from families with HDGC, Peutz-Jeghers syndrome and juvenile polyposis syndrome.

Here, we have presented the outcomes of CPG testing for 30 years in the MCGM. This has been a period of significant advances in genomic technologies and the management of families with a CPG variant, in terms of both cancer prevention and targeted therapeutic strategies. Looking ahead the challenges will be different. Whilst much of the data presented here refers to intragenic variants and (multi) exon CNVs, with ongoing advances in genetic technologies it is likely that further families will be identified with alternate means of CPG disruption, for example, variants affecting regulatory regions [30] or large structural variants [31, 32]. With the mainstreaming of diagnostic testing where immediate treatment decisions are required, robust pathways will be needed to ensure at-risk family members are offered appropriate follow up [22, 29]. It is notable that the detection of 2000 index cases in the MCGM has led to the subsequent identification of over 3000 positive family members, who can then benefit from cancer prevention and early detection strategies. Where genetic testing is undertaken in wider settings rather than a dedicated genetics clinic, accurate variant classification will be critical [33], along with attention to possible mosaicism [34] and appropriate clinical management being put in place, especially where potentially discordant clinical features and germline CPG variants are detected.

## Supplementary information

BRCA1 and BRCA2 non-index tests by family. MCGM, 1990–2020.

## Data Availability

The data sets generated and/or analysed during the current study are available from the corresponding author on reasonable request.

## References

[1] Maddock IR, Moran A, Maher ER, Teare MD, Norman A, Payne SJ (1996). A genetic register for von Hippel-Lindau disease. J Med Genet.

[2] Evans DG, Maher ER, Macleod R, Davies DR, Craufurd D (1997). Uptake of genetic testing for cancer predisposition. J Med Genet.

[3] Wonderling D, Hopwood P, Cull A, Douglas F, Watson M, Burn J (2001). A descriptive study of UK cancer genetics services: an emerging clinical response to the new genetics. Br J Cancer.

[4] Plon SE, Eccles DM, Easton D, Foulkes WD, Genuardi M, Greenblatt MS (2008). IARC Unclassified Genetic Variants Working Group. Sequence variant classification and reporting: recommendations for improving the interpretation of cancer susceptibility genetic test results. Hum Mutat.

[5] Richards S, Aziz N, Bale S, Bick D, Das S, Gastier-Foster J (2015). Standards and guidelines for the interpretation of sequence variants: a joint consensus recommendation of the American College of Medical Genetics and Genomics and the Association for Molecular Pathology. Genet Med.

[6] Malkin D, Li FP, Strong LC, Fraumeni JF, Nelson CE, Kim DH (1990). Germ line p53 mutations in a familial syndrome of breast cancer, sarcomas, and other neoplasms. Science.

[7] Rahman N, Seal S, Thompson D, Kelly P, Renwick A, Elliott A (2007). PALB2, which encodes a BRCA2-interacting protein, is a breast cancer susceptibility gene. Nat Genet.

[8] Antoniou AC, Casadei S, Heikkinen T, Barrowdale D, Pylkäs K, Roberts J (2014). Breast-cancer risk in families with mutations in PALB2. N Engl J Med.

[9] Yang X, Leslie G, Doroszuk A, Aittomäki K, Blomqvist C, Heikkinen T et al. Cancer risks associated with germline PALB2 pathogenic variants: an international study of 524 families. J Clin Oncol. 2020;38:674–85. 10.1200/JCO.19.01907 PMC7049229 31841383

[10] Rebbeck TR, Burns-White K, Chan AT, Emmons K, Freedman M, Hunter DJ (2018). Precision prevention and early detection of cancer: fundamental principles. Cancer Discov.

[11] Kaufman B, Shapira-Frommer R, Schmutzler RK, Audeh MW, Friedlander M, Balmaña J (2015). Olaparib monotherapy in patients with advanced cancer and a germline BRCA1/2 mutation. J Clin Oncol.

[12] Le DT, Uram JN, Wang H, Bartlett BR, Kemberling H, Eyring AD (2015). PD-1 blockade in tumors with mismatch-repair deficiency. N Engl J Med.

[13] Vasen HF, Mecklin JP, Khan PM, Lynch HT (1991). The international collaborative group on hereditary non-polyposis colorectal cancer (ICG-HNPCC). Dis Colon Rectum.

[14] Vasen HF, Watson P, Mecklin JP, Lynch HT (1999). New clinical criteria for hereditary nonpolyposis colorectal cancer (HNPCC, Lynch syndrome) proposed by the International Collaborative group on HNPCC. Gastroenterology.

[15] van der Klift HM, Tops CM, Bik EC, Boogaard MW, Borgstein AM, Hansson KB (2010). Quantification of sequence exchange events between PMS2 and PMS2CL provides a basis for improved mutation scanning of Lynch syndrome patients. Hum Mutat.

[16] Li J, Dai H, Feng Y, Tang J, Chen S, Tian X (2015). A comprehensive strategy for accurate mutation detection of the highly homologous PMS2. J Mol Diagn.

[17] Dominguez-Valentin M, Sampson JR, Seppälä TT, Sanne W, Plazzer JP, Nakken S (2020). Cancer risks by gene, age, and gender in 6350 carriers of pathogenic mismatch repair variants: findings from the Prospective Lynch Syndrome Database. Genet Med.

[18] Grzymski JJ, Elhanan G, Rosado JM, Smith E, Schlauch KA, Read R (2020). Population genetic screening efficiently identifies carriers of autosomal dominant diseases. Nat Med.

[19] Wimmer K, Kratz CP, Vasen HF, Caron O, Colas C, Entz-Werle N (2014). Diagnostic criteria for constitutional mismatch repair deficiency syndrome: suggestions of the European consortium ‘care for CMMRD’(C4CMMRD). J Med Genet.

[20] Evans DG, Eccles DM, Rahman N, Young K, Bulman M, Amir E (2004). A new scoring system for the chances of identifying a BRCA1/2 mutation outperforms existing models including BRCAPRO. J Med Genet.

[21] Evans DG, Harkness EF, Plaskocinska I, Wallace AJ, Clancy T, Woodward ER (2017). Pathology update to the Manchester Scoring System based on testing in over 4000 families. J Med Genet.

[22] Flaum N, Morgan RD, Burghel GJ, Bulman M, Clamp AR, Hasan J (2020). Mainstreaming germline BRCA1/2 testing in non-mucinous epithelial ovarian cancer in the North West of England. Eur J Hum Genet.

[23] Dorling L, Carvalho S, Allen J, González-Neira A, Luccarini C, Breast Cancer Association Consortium (2021). Breast cancer risk genes: association analysis in more than 113,000 women. N Engl J Med.

[24] Foulkes WD, Smith IE, Reis-Filho JS (2010). Triple-negative breast cancer. N Engl J Med.

[25] Morgan RD, Burghel GJ, Flaum N, Bulman M, Clamp AR, Hasan J (2019). Prevalence of germline pathogenic BRCA1/2 variants in sequential epithelial ovarian cancer cases. J Med Genet.

[26] Hampel H, de la Chapelle A (2013). How do we approach the goal of identifying everybody with Lynch Syndrome?. Fam Cancer.

[27] Smith MJ, Urquhart JE, Harkness EF, Miles EK, Bowers NL, Byers HJ (2016). The contribution of whole gene deletions and large rearrangements to the mutation spectrum in inherited tumor predisposing syndromes. Hum Mutat.

[28] Shendure J, Porreca GJ, Reppas NB, Lin X, McCutcheon JP (2005). Accurate multiplex polony sequencing of an evolved bacterial genome. Science.

[29] Menko FH, Jeanson KN, Bleiker EM, van Tiggelen CW, Hogervorst FB, ter Stege JA (2020). The uptake of predictive DNA testing in 40 families with a pathogenic BRCA1/BRCA2 variant. An evaluation of the proband-mediated procedure. Eur J Hum Genet.

[30] Evans DG, van Veen EM, Byers HJ, Wallace AJ, Ellingford JM, Beaman G (2018). A dominantly inherited 5′ UTR variant causing methylation-associated silencing of BRCA1 as a cause of breast and ovarian cancer. Am J Hum Genet.

[31] Hyder Z, Fairclough A, Groom M, Getty J, Alexander E, van Veen EM et al. Constitutional de novo deletion CNV encompassing REST predisposes to diffuse hyperplastic perilobar nephroblastomatosis (HPLN). J Med Genet. 2020;58:581–5. 10.1136/jmedgenet-2020-107087 32917767

[32] Thibodeau ML, O’Neill K, Dixon K, Reisle C, Mungall KL, Krzywinski M (2020). Improved structural variant interpretation for hereditary cancer susceptibility using long-read sequencing. Genet Med.

[33] Garrett A, Callaway A, Durkie M, Cubuk C, Alikian M, Burghel GJ (2020). Cancer Variant Interpretation Group UK (CanVIG-UK): an exemplar national subspecialty multidisciplinary network. J Med Genet.

[34] Evans DG, Woodward ER (2021). New surveillance guidelines for Li-Fraumeni and hereditary TP53 related cancer syndrome: implications for germline TP53 testing in breast cancer. Fam Cancer.

