# Supplementary material for: 30 year experience of index case identification and outcomes of cascade testing in high-risk breast and colorectal cancer predisposition genes
Source: Eur J Hum Genet. 2021 Dec 6;30(4):413–9. doi: 10.1038/s41431-021-01011-8 (PMC8645350; doi:10.1038/s41431-021-01011-8)
Supplement: Supplementary file 1 — BRCA1 and BRCA2 non-index tests by family. MCGM, 1990–2020. [file 41431_2021_1011_MOESM1_ESM.docx]

**Supplementary Table 1. *BRCA1* and *BRCA2* non-index tests by family. MCGM, 1990-2020.**

|  | ***BRCA1*** | | ***BRCA2*** | |
| --- | --- | --- | --- | --- |
|  | **Minimum** | **Maximum** | **Minimum** | **Maximum** |
| **Tests in females per family** |  | | | |
| Number of tests with positive result | 0 | 8 | 0 | 20 |
| Number of tests with negative result | 0 | 18 | 0 | 39 |
| *Total tests undertaken | 0 | 23 | 0 | 59 |
| **Tests in males in a family** |  | | | |
| Number of tests with positive result | 0 | 5 | 0 | 8 |
| Number of tests with negative result | 0 | 4 | 0 | 7 |
| *Total tests undertaken | 0 | 8 | 0 | 15 |
| **All tests in a family** |  |  |  |  |
| Number of tests with positive result | 0 | 12 | 0 | 28 |
| Number of tests with negative result | 0 | 19 | 0 | 36 |
| *Total tests undertaken | 0 | 25 | 0 | 74 |
|  |  | | | |
|  | **Families with this number of non-index positive tests (%)** | | | |
|  | ***BRCA1*** | | ***BRCA2*** | |
|  | **Tests in females** | **Tests in males** | **Tests in females** | **Tests in males** |
| **Number of non-index positive tests** |  | | | |
| 0 | 44.71 | 73.56 | 42.46 | 70.41 |
| 1-2 | 44.71 | 23.87 | 44.08 | 27.51 |
| 3-5 | 9.21 | 2.57 | 12.72 | 1.92 |
| 6-10 | 1.36 | 0.00 | 0.59 | 0.15 |
| >10 | 0.00 | 0.00 | 0.15 | 0.00 |
| ^#^Total | 100.00 | 100.00 | 99.85 | 100.00 |

*Numbers for totals are not necessarily a direct sum as each individual family will have had a variable number of non-index tests undertaken, for example a single family will not necessarily have had the maximum number of tests in females and maximum number of tests in males.

^#^ Not all totals are not exactly 100% due to rounding errors.
